# Supplementary material for: Strong plasmon-molecule coupling at the nanoscale revealed by first-principles modeling
Source: Nat Commun. 2019 Jul 26;10:3336. doi: 10.1038/s41467-019-11315-5 (PMC6659639; doi:10.1038/s41467-019-11315-5)
Supplement: Supplementary file 1 — Supplementary information for publication [file 41467_2019_11315_MOESM1_ESM.pdf]

Supplementary Information

Strong plasmon-molecule coupling at the nanoscale revealed by first-principles modeling

Rossi *et al.*

Contents

Supplementary Figures 2
1. Visualization of structures . . . 2
2. Photoabsorption spectra of benzene molecules . . . 3
3. Density of states of benzene molecules . . . 3
4. Transition contribution maps of all nanoparticles . . . 4
5. Shift of the plasmon resonance caused by benzene molecules . . . 4
6. Fitting results for Al_{201} coupled to benzene molecules . . . 5
7. Fitting results for Al_{586} coupled to benzene molecules . . . 5
8. Fitting results for Al_{1289} coupled to benzene molecules . . . 6
9. Fitting results for Al_{201} coupled to two benzene molecules with varying distance . . 7

Supplementary Tables 8
1. Fitting parameters for Al_{201} coupled to benzene molecules . . . 8
2. Fitting parameters for Al_{586} coupled to benzene molecules . . . 8
3. Fitting parameters for Al_{1289} coupled to benzene molecules . . . 8
4. Fitting parameters for Al_{201} coupled to two benzene molecules with varying distance 9

Supplementary Notes 9
1. Fitting of absorption spectra . . . 9

Supplementary Figures

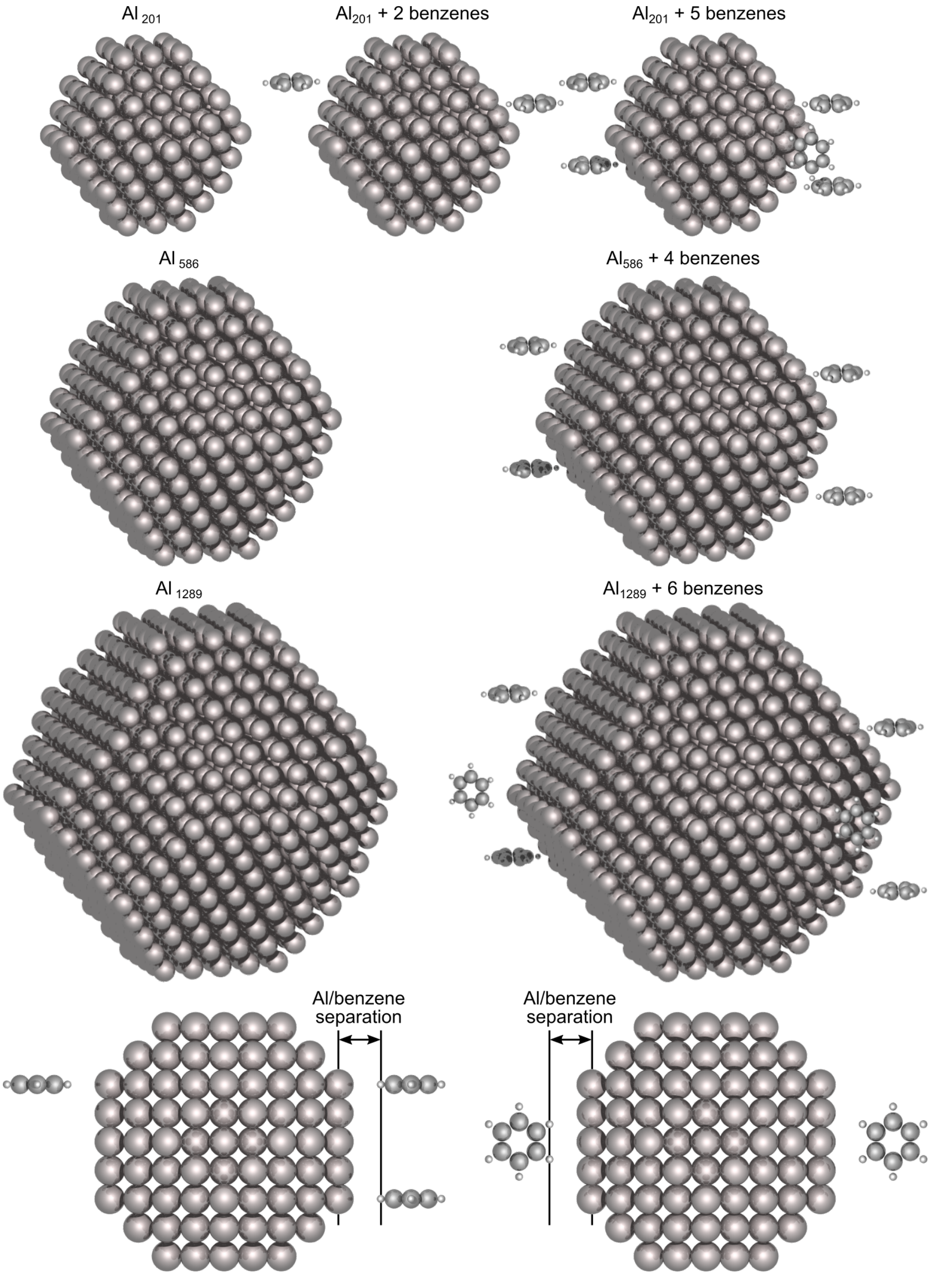

Supplementary Figure 1: Visualization of structures. Examples of the modeled aluminum regular truncated octahedra (Al_{201}, Al_{586}, Al_{1289}) coupled to the benzene molecules are shown. The molecules are placed symmetrically at the corners of the nanoparticle so that the molecules are subject to an as-identical-as-possible induced electric field. The molecules are added alternately to the sides of the nanoparticle so that either side of the particle has at most one benzene molecule more than the other side. The molecules are aligned parallel to the direction of the external electric field. The Al/benzene separation is defined as the closest distance between the hydrogen atoms and the plane of the Al facet.

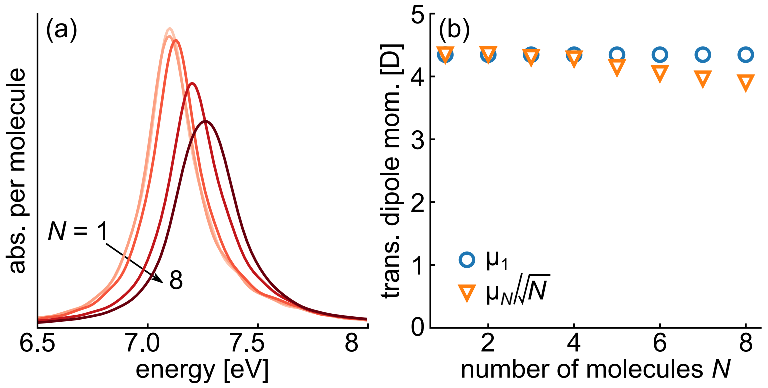

Supplementary Figure 2: Photoabsorption spectra of benzene molecules. (a) Photoabsorption spectra of benzene molecules for $N = 1, 2, 4, 6, 8$. The molecules are placed in the same configuration as when coupled to the Al_{201} particle. (b) The effective transition dipole moment $\mu_N/\sqrt{N}$ per molecule calculated from the spectra. Due to the small distance between the molecules, the coupling between the molecules themselves causes a slight shift in their photoabsorption spectra, and the effective transition dipole moment decreases up to 10% for $N = 8$. For $N \leq 4$ the spectra are almost unchanged, indicating negligible coupling between molecules. In the larger nanoparticles with larger facets, such changes are smaller due to the larger molecule-molecule separation.

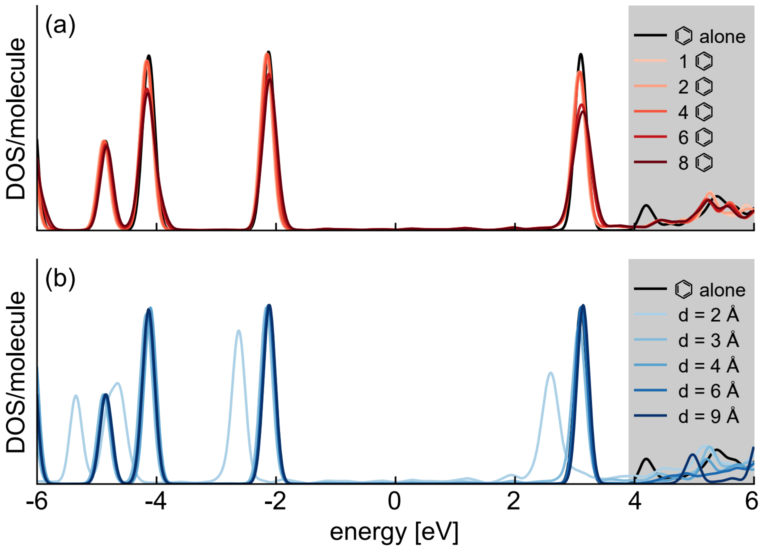

Supplementary Figure 3: Density of states of benzene molecules. The projected DOS of benzene molecules coupled to the Al_{201} particle (a) as the number of molecules is increased and (b) as the nanoparticle-molecule separation d is varied. The shaded region indicates energies above the vacuum level ($\sim$4 eV), which is not fully captured by local basis sets. Only small changes are seen up to the lowest unoccupied molecular orbital (LUMO) and the energy difference between LUMO and the highest occupied molecular orbital (HOMO) is similar for all considered cases, except when the separation decreases below 3 Å.

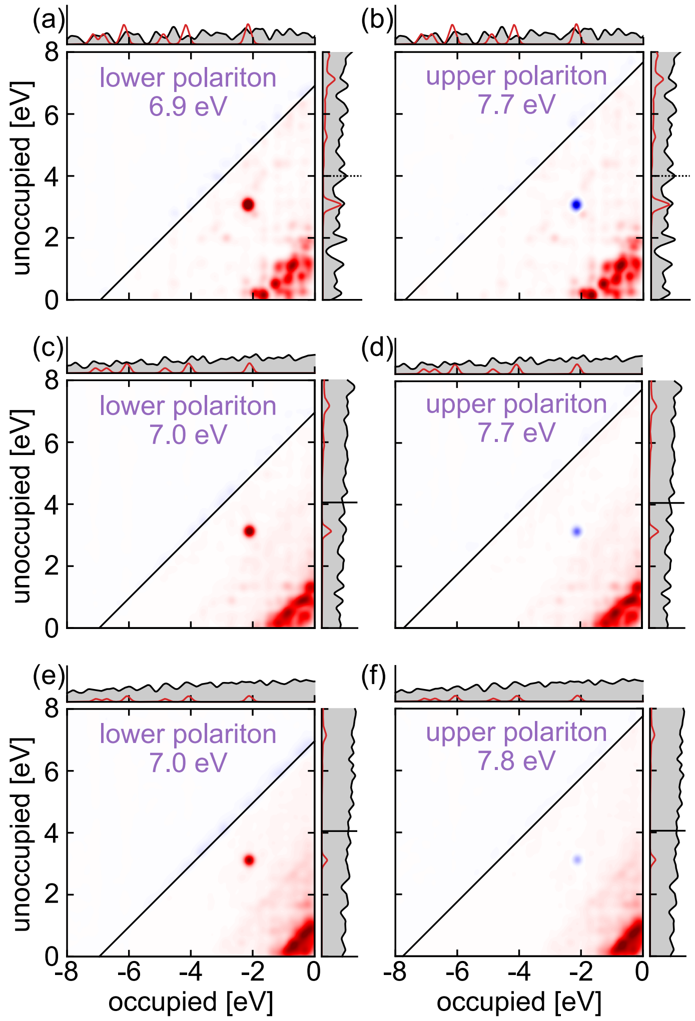

Supplementary Figure 4: Transition contribution maps of all nanoparticles. TCMs for all calculated nanoparticles with two benzene molecules. The left and right columns correspond to the lower and upper polaritons, respectively. (a,b) Al_{201}, (c,d) Al_{586}, (e,f) Al_{1289}.

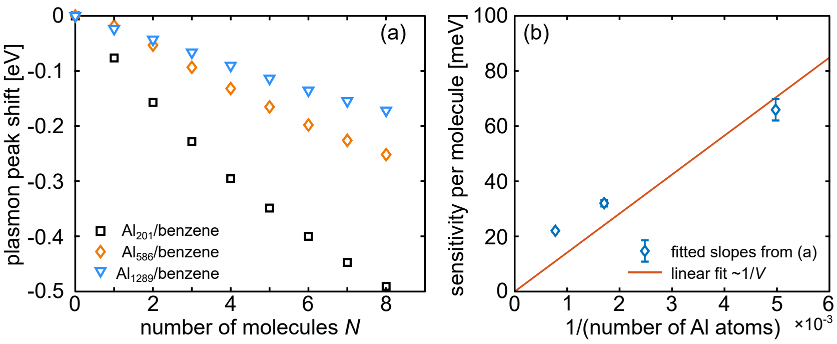

Supplementary Figure 5: Shift of the plasmon resonance caused by benzene molecules. (a) Plasmon peak shift induced by the presence of benzene molecules for the three Al nanoparticles as given by ω_{pl} in Supplementary Tables 1–3. (b) Plasmon peak shift (sensitivity) per molecule is approximately inversely proportional to the nanoparticle volume V (which is proportional to the number of Al atoms). The errorbars mark the fitting accuracy of the slopes in panel (a).

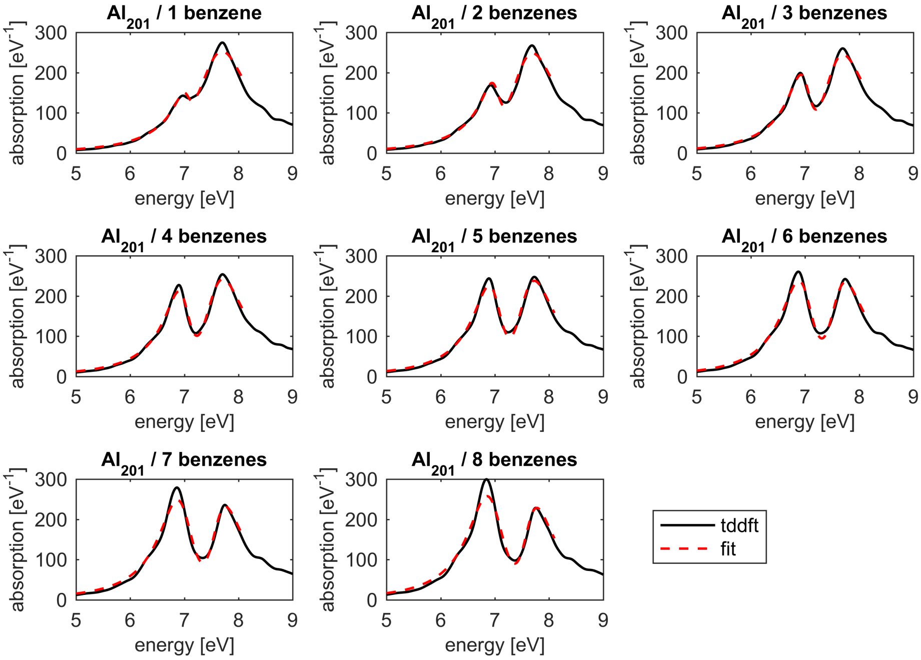

Supplementary Figure 6: Fitting results for Al_{201} coupled to benzene molecules. The fits correspond to Equation (1) with parameters given by Supplementary Table 1.

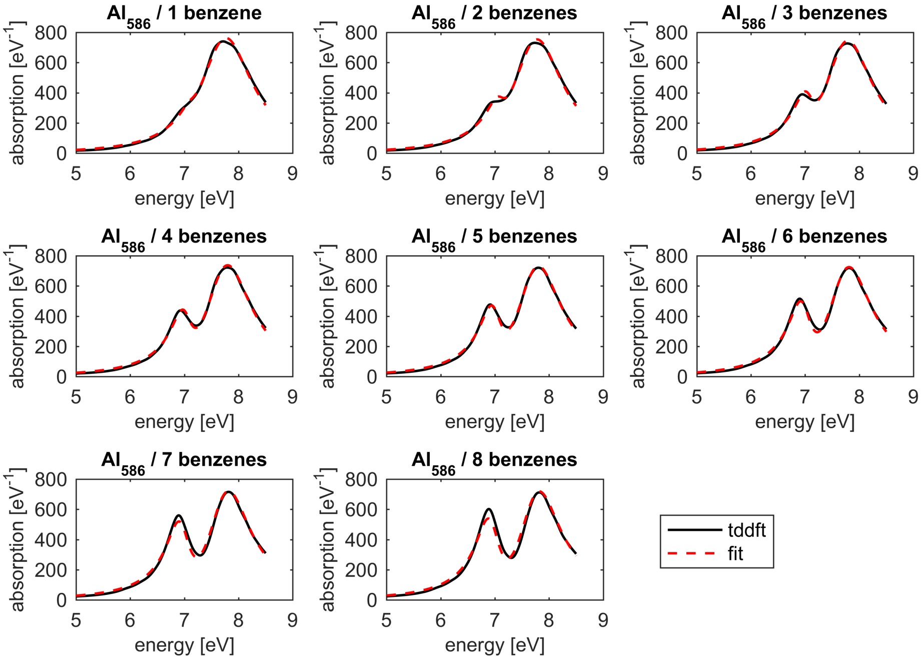

Supplementary Figure 7: Fitting results for Al_{586} coupled to benzene molecules. The fits correspond to Equation (1) with parameters given by Supplementary Table 2.

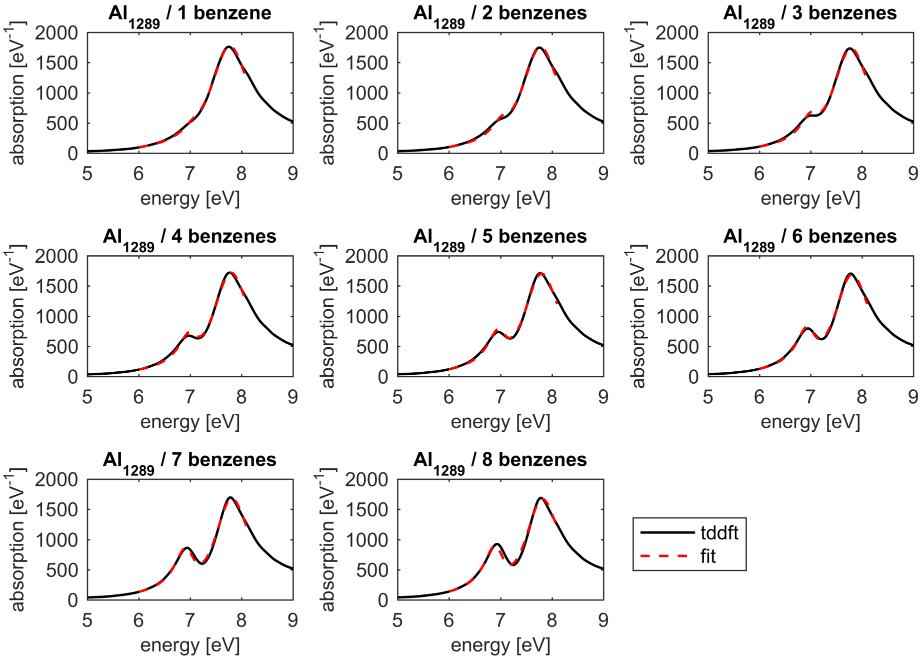

Supplementary Figure 8: Fitting results for Al_{1289} coupled to benzene molecules. The fits correspond to Equation (1) with parameters given by Supplementary Table 3.

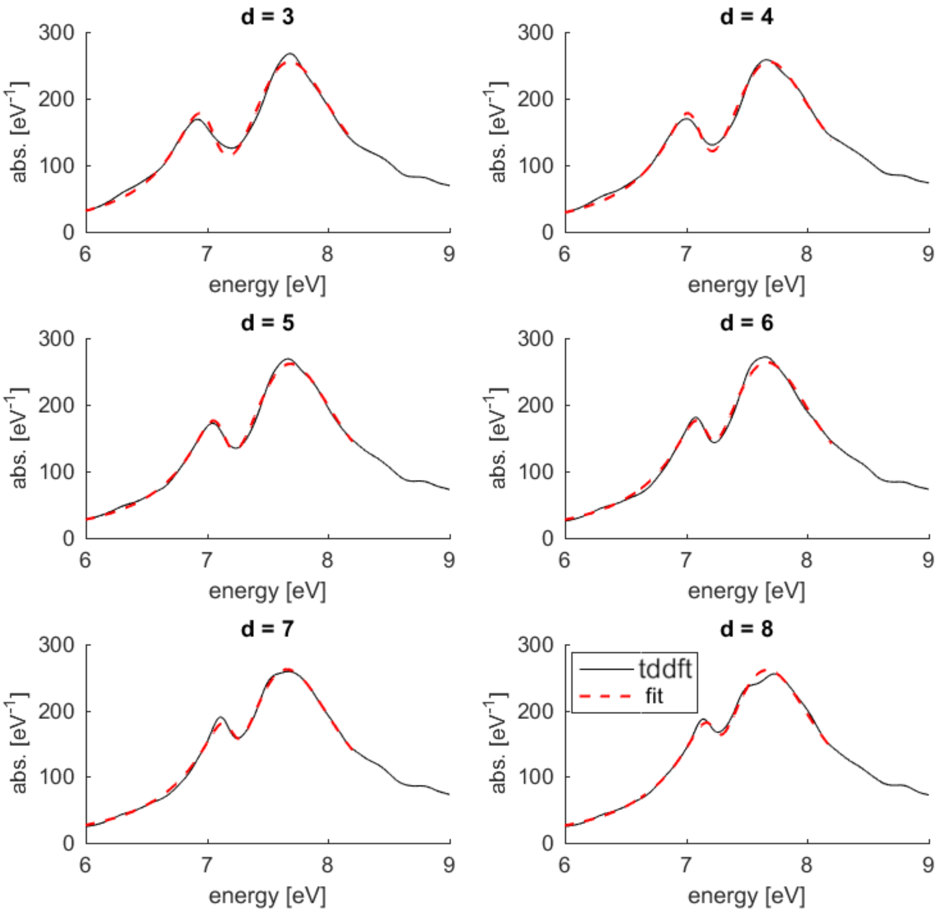

Supplementary Figure 9: Fitting results for Al_{201} coupled to two benzene molecules with varying distance. The fits correspond to Equation (1) with parameters given by Supplementary Table 4.

Supplementary Tables

Supplementary Table 1: Fitting parameters for Al_{201} coupled to benzene molecules. The fitted plasmon width of the bare Al nanoparticle is $\gamma_{pl} = 1.2\,eV$ and the fitted exciton width of the bare benzene molecule is $\gamma_{ex} = 0.3\,eV$. These values are fixed in the fitting of the coupled spectra. See Supplementary Note 1 for description of the parameters.

N	a [arb. units]	ω_{pl} [eV]	ω_{ex} [eV]	g [eV]
–	320	7.66		
1	337	7.58	7.10	0.202
2	339	7.50	7.13	0.275
3	344	7.43	7.17	0.324
4	349	7.36	7.21	0.361
5	354	7.31	7.26	0.380
6	359	7.26	7.31	0.396
7	366	7.21	7.35	0.407
8	373	7.17	7.39	0.416

Supplementary Table 2: Fitting parameters for Al_{586} coupled to benzene molecules. The fitted plasmon width of the bare Al nanoparticle is $\gamma_{pl} = 1.2\,eV$ and the fitted exciton width of the bare benzene molecule is $\gamma_{ex} = 0.3\,eV$. These values are fixed in the fitting of the coupled spectra. See Supplementary Note 1 for description of the parameters.

N	a [arb. units]	ω_{pl} [eV]	ω_{ex} [eV]	g [eV]
–	914	7.74		
1	919	7.72	7.13	0.106
2	923	7.69	7.13	0.191
3	929	7.65	7.14	0.255
4	935	7.61	7.14	0.303
5	941	7.58	7.15	0.337
6	947	7.54	7.16	0.366
7	953	7.51	7.17	0.389
8	961	7.49	7.18	0.409

Supplementary Table 3: Fitting parameters for Al_{1289} coupled to benzene molecules. The fitted plasmon width of the bare Al nanoparticle is $\gamma_{pl} = 1.0\,eV$, and the fitted exciton width and resonance energy of the bare benzene molecule are $\gamma_{ex} = 0.3\,eV$ and $\omega_{ex} = 7.1\,eV$, respectively. These values are fixed in the fitting of the coupled spectra. See Supplementary Note 1 for description of the parameters.

N	a [arb. units]	ω_{pl} [eV]	ω_{ex} [eV]	g [eV]
–	1730	7.76		
1	1729	7.74	7.10	0.103
2	1735	7.72		0.166
3	1743	7.69		0.216
4	1752	7.67		0.257
5	1760	7.65		0.291
6	1768	7.63		0.320
7	1775	7.61		0.344
8	1783	7.59		0.364

Supplementary Table 4: Fitting parameters for Al_{201} coupled to two benzene molecules with varying distance. The fitted plasmon width of the bare Al nanoparticle is $\gamma_{\mathrm{pl}} = 1.2\,\mathrm{eV}$. This value is fixed in the fitting of the coupled spectra. See Supplementary Note 1 for description of the parameters.

distance [Å]	a [arb. units]	ω_{pl} [eV]	ω_{ex} [eV]	γ_{ex} [eV]	g [eV]
–	320	7.66			
3	326	7.49	7.13	0.25	0.283
4	321	7.52	7.17	0.23	0.244
5	326	7.55	7.19	0.22	0.212
6	326	7.56	7.20	0.21	0.177
7	324	7.57	7.23	0.21	0.157
8	322	7.58	7.26	0.21	0.145

Supplementary Notes

Supplementary Note 1: Fitting of absorption spectra. The photoabsorption spectra $\sigma_{\mathrm{abs}}(\omega)$ of the coupled nanoparticle/molecule systems were fitted using a coupled oscillator model

$$\sigma_{\mathrm{abs}}(\omega) = a\omega \mathrm{Im}\left[\frac{\omega_{\mathrm{ex}}^2 - (\omega + i\gamma_{\mathrm{ex}}/2)^2}{\left(\omega_{\mathrm{pl}}^2 - (\omega + i\gamma_{\mathrm{pl}}/2)^2\right)\left(\omega_{\mathrm{ex}}^2 - (\omega + i\gamma_{\mathrm{ex}}/2)^2\right) - 4g^2\omega^2}\right], \quad (1)$$

where the fitting parameters are amplitude a, plasmon resonance energy and width ω_{pl} and γ_{pl}, exciton resonance energy and width ω_{ex} and γ_{ex}, and the coupling strength g.

The fitting was carried out via two approaches: one-by-one or globally. In the first one-by-one approach, the spectra of coupled systems were fitted independently to Eq. (1) with a, ω_{pl}, ω_{ex}, and g as free fitting parameters while keeping γ_{pl} and γ_{ex} fixed to the values obtained by fitting single Lorentzian functions to the spectra of bare nanoparticle and benzene molecule. The results of these fittings are shown in Supplementary Tables 1–4 and Supplementary Figures 6–9, and they are used in the analysis in the article. In the case of varied nanoparticle/molecule separation, γ_{ex} was kept as a free fitting parameter, which results in slightly different parameters in Supplementary Tables 1 (for $N = 2$) and 4 (for 3 Å) although the system is the same in both cases.

In the second global-fitting approach, the fitting was performed to a two-argument function $\sigma_{\mathrm{abs}}(\omega, N)$ for each nanoparticle size to confirm the results obtained from the above approach. For this purpose, we assumed the following functional forms for the parameters of Eq. (1): 1) ω_{pl} red shifts linearly with N, 2) γ_{pl} is constant with N, 3) ω_{ex} changes with N due to the mutual interaction between molecules as in Supplementary Figure 2 for Al_{201} (for Al_{1289} the effect is small and a constant ω_{ex} is assumed), 4) γ_{ex} is constant with N, 5) g increases as $\sqrt{N}$ with N (assuming equal coupling strength to each molecule) or, in the case of varied separation, g decreases with distance with a power dependence. This global fitting results in good quantitative agreement with the calculated spectra and agree well with the results of the individual fits from the first approach.
